# Interpreting Outcome Data in Hematological Malignancies: A Paradigm for Clinical Studies

**DOI:** 10.5041/RMMJ.10104

**Published:** 2013-01-30

**Authors:** Jacob M. Rowe

**Affiliations:** Department of Hematology, Shaare Zedek Medical Center, Jerusalem, Israel; Department of Hematology and Bone Marrow Transplantation, Rambam Medical Center, Haifa, Israel; and Technion, Israel Institute of Technology, Haifa, Israel

**Keywords:** Bone marrow transplantation, hematological malignancies, leukemia, lymphoma, outcome data

## Abstract

Results of clinical studies are often contradictory in real time, and in other instances therapies may be adopted due to information from clinical studies where the data may be premature or resulting from small studies. Much of the data may have inherent selection biases, and their interpretation may be confusing and difficult. The hematological literature is full of such examples, and this review will describe some such instances in the hope of introducing both a cautionary note and encouraging more precise description of study conditions as well as an appreciation of the importance of allowing data from clinical studies to mature. Several examples will be drawn from clinical studies in lymphomas, leukemia, and bone marrow transplantation.

## LYMPHOMA

### Diffuse Large Cell Lymphoma

In the mid-1970s the standard of care for the treatment of diffuse large cell lymphoma (or diffuse histiocytic lymphoma, as it was then known) was a combination of cyclophosphamide, doxorubicin, vincristine, and prednisone. This, or a modified version of these drugs, known as CHOP, initially developed at the National Cancer Institute in the US in the mid-1970s,^1^ was generally given every 3 weeks for six cycles, and this was the historic standard of care for lymphoma, with reported survivals of 35%–40%. In the late 1970s and in the early 1980s, following the work of Norton and Simon^2^ in 1977 and Goldie and Coldman^3^ in 1982, many of the advances in the design of cancer studies followed the Goldie–Coldman hypothesis which, in essence, described the necessity for considering the intensity, timing, and the use of alternating non-cross-resistant drugs as critical for the success of cancer therapy. As a result of these studies multiple new regimens were reported in the early 1980s with second-generation treatments for lymphoma which included the acronyms COP-BLAM, m-BACOD, M-BACOD with reported survivals of 55%–60%. These were followed by the third-generation regimens for the treatment of diffuse large cell lymphomas, including combinations such as ProMACE-MOPP, COP-BLAM III, ProMACE-CytaBOM, and MACOP-B with reported overall survivals of 65%–75% (Table 1). The reports from these second and third generations were so astonishing that many considered the “historic” standard of CHOP to be unethical. An editorial in the *Annals of Internal Medicine* in 1985 concluded that “the results of second- and third-generation chemotherapy regimens are so consistently good from so many independent sources, that they continue to engender even more ferment in the treatment of large cell lymphoma.”^4^

**Figure 1:**
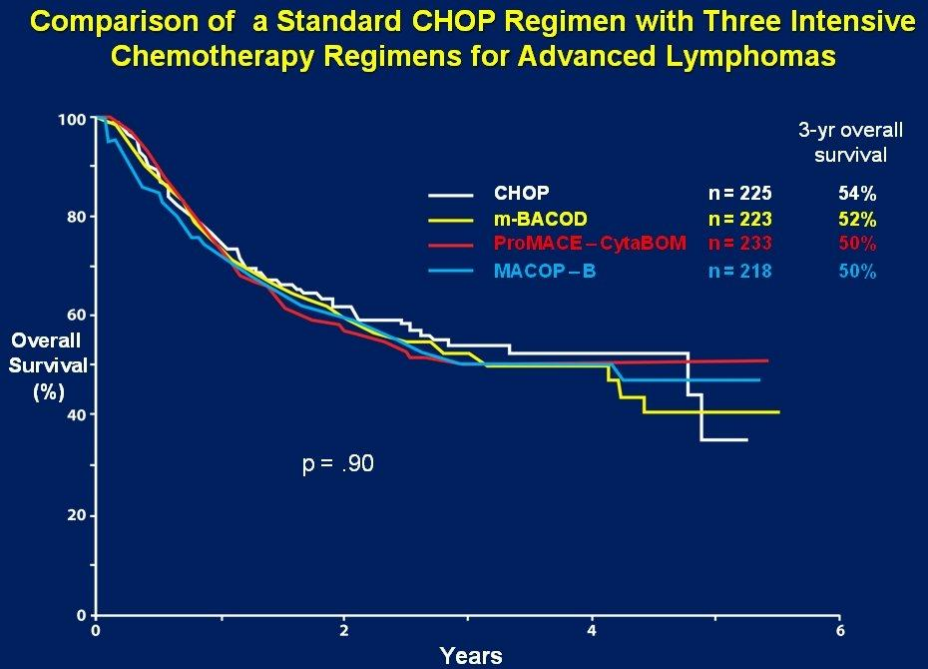
**Overall survival of CHOP regimen prospectively compared with three third-generation regimens.** Published with permission from Fisher RI, et al. Comparison of a standard regimen (CHOP) with three intensive chemotherapy regimens for advanced non-Hodgkin’s lymphoma. N Engl J Med 1993;328(14):1002–6.^5^

Against this general background, in the late 1980s, the Southwest Oncology Group and the Eastern Oncology Group in the US initiated a prospective randomized phase III trial comparing the standard CHOP regimen with three intensive chemotherapy regimens for advanced lymphomas. The results published in the *New England Journal of Medicine* in 1993 astounded the hematology community with similar overall survival for all regimens and with no subgroup of patients in which survival was improved by a third-generation regimen (Figure 1).^5^ Furthermore, the CHOP regimen was less toxic, thus concluding that CHOP remained the best available treatment for patients with advanced-stage intermediate- or high-grade lymphomas. These remarkable results highlighted the *difficulty of interpreting limited phase II data due to inherent selection biases*. To this day CHOP remains the standard of care for aggressive lymphomas and is the yard-stick against which all new advances are compared. The only proven advance in the management of lymphoma has been the addition of rituximab which was established through a carefully controlled phase III study where CHOP alone was the comparator arm.^6^

### Relapsed Aggressive Lymphoma

**Figure 2:**
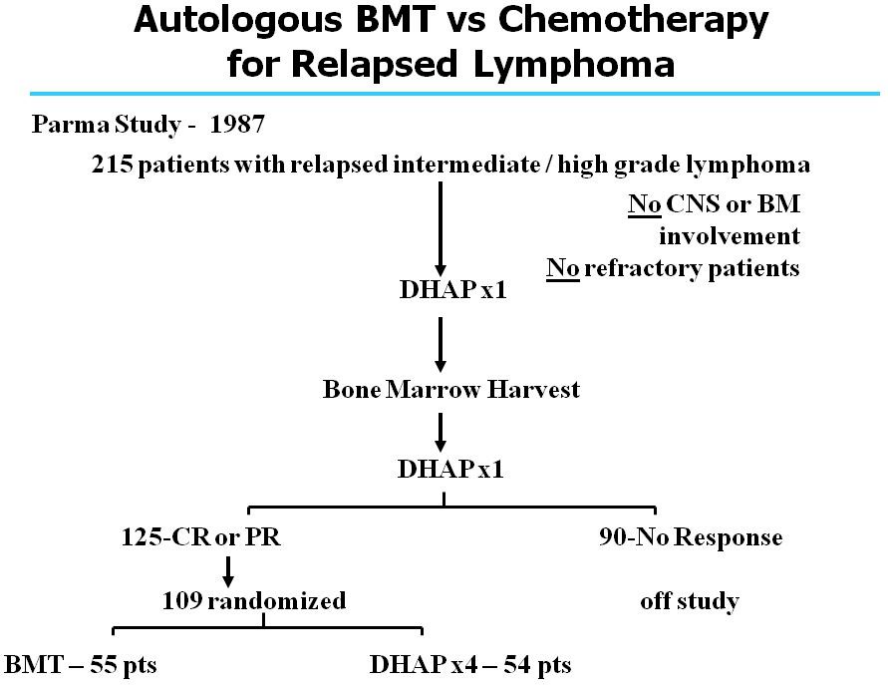
**Schematic design of the PARMA study.** Published with permission from Philip T, et al. Autologous bone marrow transplantation as compared with salvage chemotherapy in relapses of chemotherapy-sensitive non-Hodgkin’s lymphoma. N Engl J Med 1995;333:1540–5.^20^

**Figure 3:**
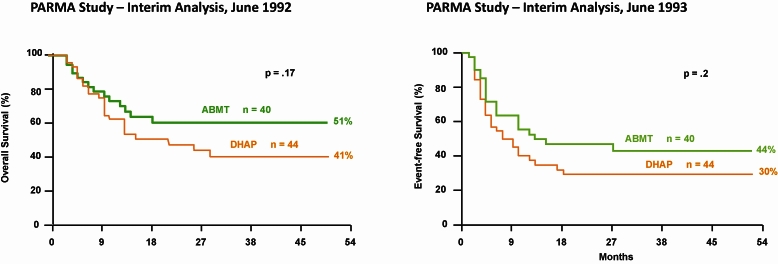
**Preliminary data from the PARMA study, as presented at international meetings in 1992 and 1993.**

**Figure 4:**
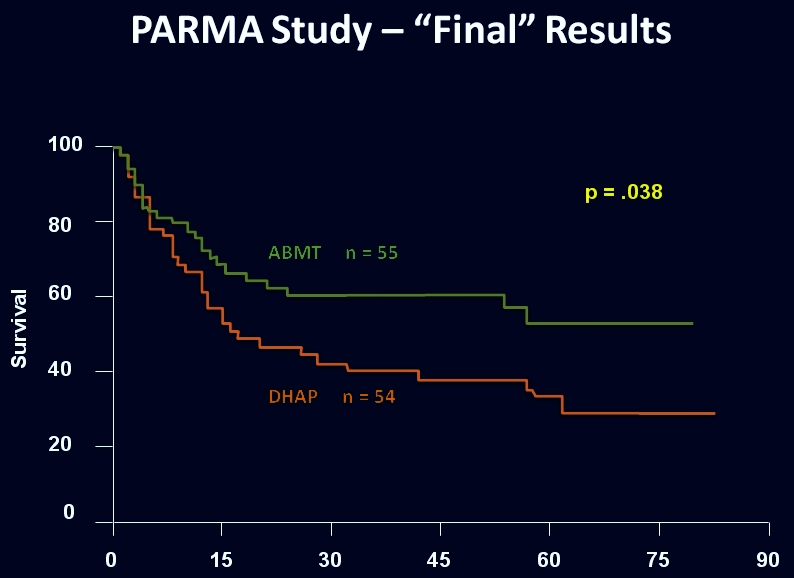
**Overall survival of patients randomized to either high-dose therapy followed by transplantation or conventional therapy.** Published with permission from Philip T, et al. Autologous bone marrow transplantation as compared with salvage chemotherapy in relapse of chemotherapy-sensitive non-Hodgkin’s lymphoma. N Engl J Med 1995;333:1540–5.^20^

Another example relates to the management of relapsed aggressive lymphomas. Early data in the 1980s suggested that the results from autologous transplantation were far superior to the use of traditional conventional chemotherapy, which in fact yielded almost no cures for the disease. Nevertheless, given the lessons learned from the phase III study of CHOP, some skepticism existed in the hematologic community, and the need for a prospective phase III study was clearly apparent. The PARMA study (Figure 2) was designed specifically for this purpose in 1987. Recruitment was difficult due to a reluctance by many practitioners to offer standard chemotherapy to even those with the better prognosis among the relapsed groups. Preliminary data, presented at international meetings in 1992 and 1993 (Figure 3), were widely interpreted as demonstrating that high-dose therapy with autologous transplantation did not provide a significant improvement. This created quite a stir in the transplant community until the definitive results from the trial were published in the *New England Journal of Medicine* in 1995, demonstrating that, compared with conventional chemotherapy, treatment with high-dose chemotherapy followed by autologous bone marrow transplantation increases the survival in patients with chemotherapy-sensitive relapsed lymphoma (Figure 4).

## ACUTE MYELOID LEUKEMIA (AML)

### Complete Remission

**Figure 5:**
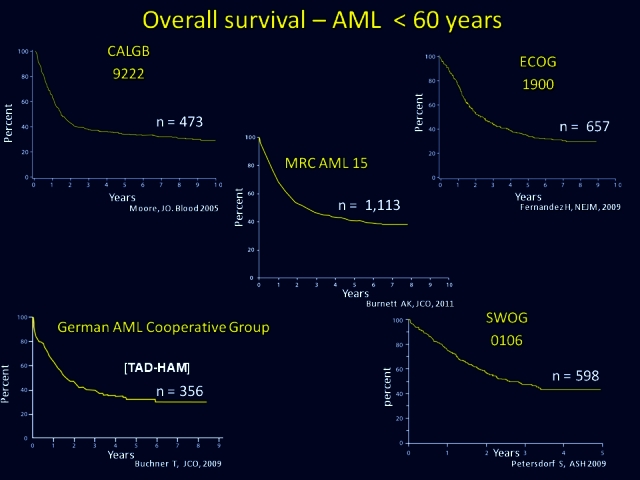
**Overall survival from diagnosis of patients younger than 60 years with acute myeloid leukemia.** Curves are practically identical despite variations in treatment regimens, countries, trial period, population size, and co-operative group. Results from CALGB 9222^21^; ECOG 1900^22^; German AML Cooperative Group^23^; SWOG 0106^24^; and MRC AML 15.^25^ Published with permission from Rowe JM. Evaluation of prognostic factors in AML. Best Pract Res Clin Haematol 2011;24:485–8.

Although it has been known for a long time that achieving a complete remission is the *sine qua non* for long-term survival, induction of remission has been fairly standardized over the past four decades. Standard induction for AML consists of 3 days of an anthracycline, usually daunorubicin, together with 7 days of cytarabine. The problem here relates to data published in the late 1980s and the 1990s, which indicated that using virtually identical drug regimens the complete remission rate varied from 55% to 60% among the Southwest Oncology Group (SWOG) in the US, 65%–70% among the Eastern Cooperative Oncology Group (ECOG) in the US, 70%–75% in the Cancer and Leukemia Group B (CALGB) in the US, and 75%–85% in Medical Research Council (MRC) in Britain (Table 2). Despite these differences in the complete remission rate, the overall outcome for AML for younger adults is virtually identical in each of the major groups when evaluating for survival from diagnosis (Figure 5).^7^ The question still remained how these identical survival results could be achieved when there are such heterogeneous reports of the complete remission rates. Although not always clearly specified in the manuscripts, it was clear to practitioners that these discrepancies did not reflect an inherent difference in practice or responses within institutions. The explanation here reflects a *difference in the requirement or definition of a complete response* such that, for example, in SWOG, patients needed to undergo central review at diagnosis and upon recovery of blood counts in order to confirm a complete remission. In ECOG, although central review was not required at the achievement of complete remission, final blood results needed to be performed at an ECOG-certified laboratory. This meant that if a patient was discharged from the hospital, in apparent remission, but with a platelet count of 70,000/μL, and the confirmatory platelet count of over 100,000/μL required for the definition of complete remission was not performed at an ECOG-certified laboratory, such a patient could not be categorized as achieving complete remission (Table 3).

Such subtle differences need to be clearly described in published reports to avoid either under- or over-interpretation of data.

### Phase III Studies in AML

Growth factors, granulocyte colony-stimulating factor (G-CSF) or granulocyte-macrophage colony-stimulating factor (GM-CSF), have now been demonstrated in 18 controlled studies to shorten the period of neutropenia by 4–7 days (Table 4). Despite the safety demonstrated in virtually every study, there are still physicians who hesitate using growth factors during induction therapy due to concerns for safety, related to the known increased blast cell proliferation.

The controversy has abounded for almost two decades, and one of the early negative papers for the use of cytokines was a report from the Cancer Leukemia Group B which suggested no benefit for the use of growth factors in AML. This was a well-conducted prospectively randomized study comparing GM-CSF versus placebo.^8^ However, the GM-CSF used in this study was *E. coli*-derived, a non-glycosylated GM-CSF that was highly toxic (and for this reason is no longer in clinical use). Many patients developed a rash and a fever, and the drug was discontinued during the trial period, due to safety concerns.

However, the authors correctly noted that the study drug was discontinued in one-third of patients *in each group*, presumably because the treating physician perceived that the patient had severe GM-CSF-associated toxicities, mostly rash and fever; 60/187 of patients in the GM-CSF group and 56/189 of patients in the placebo group were removed from the study.

However, what was not considered is the fact that precisely among those patients who were affected by rash or fever the GM-CSF was discontinued. Thus, the lack of benefit in the study may have reflected the fact that particularly the patients who may have benefited most from the cytokines did not receive this. The point here is to emphasize the need to *understand the precise study conditions and the caution needed in interpreting even prospectively designed placebo-controlled phase III studies*.

## ACUTE LYMPHOBLASTIC LEUKEMIA

### Lessons from Very Large Studies

The International Acute Lymphoblastic Leukemia (ALL) Study, jointly conducted by the Eastern Cooperative Oncology Group in the US and the Medical Research Council in Britain, was a large prospective study of 2,000 newly diagnosed patients with acute lymphoblastic leukemia (ALL). In this study, patients were treated identically on both sides of the Atlantic, with the data centralized in one center. ALL is a relatively uncommon disease in adults, with only approximately 1,500 new adult patients in the US per year. Because the accrual to co-operative group studies in acute leukemia in the US is no greater than 5%–10%, this means that there would be less than 150 adult patients with ALL who would be available for major co-operative group trials. These numbers make it immediately clear that in order to obtain any definitive information on this disease a national and international collaboration is needed, and this was, in fact, established in this International ALL trial. Prior to the initiation of this study in 1993, patients with standard-risk ALL were never considered for an allogeneic transplant in first complete remission. In fact, the largest trial of bone marrow transplantation prior to the international ALL study was the French LALA-94 study which was published in 2004.^9^ That study demonstrated a benefit for high-risk ALL patients who had a sibling donor over those who did not have a sibling donor. However, standard-risk patients (i.e. those patients younger than 35 years who did not have a high white cell count at presentation and who went into remission within the first 4 months) were not even studied.

**Figure 6:**
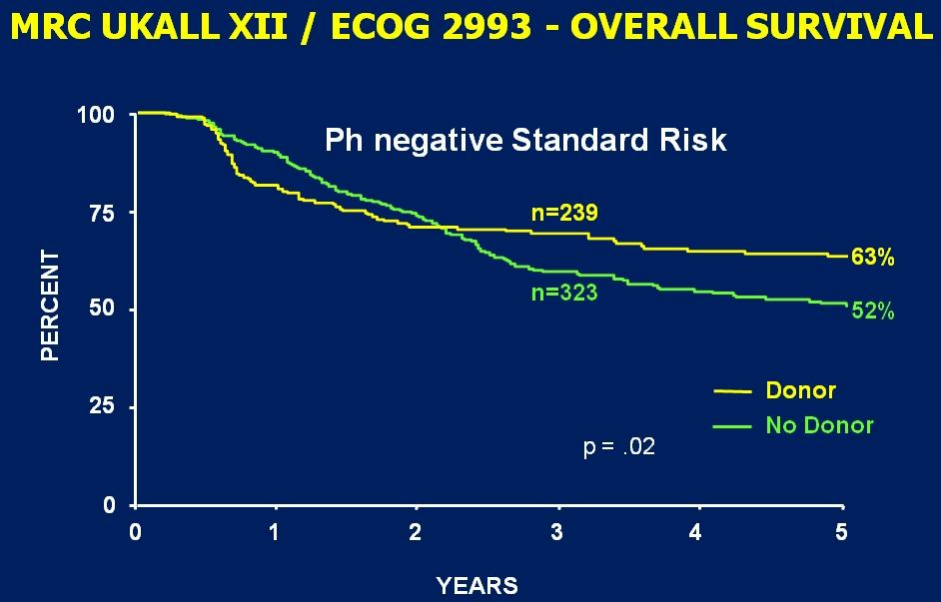
**Overall survival from diagnosis for donor versus no-donor for Ph-negative patients. Estimation of the effect of sibling donor transplant versus chemotherapy in standard-risk patients.** Published with permission from Goldstone AH, et al. In adults with standard-risk acute lymphoblastic leukemia, the greatest benefit is achieved from a matched sibling allogeneic transplantation in first complete remission, and an autologous transplantation is less effective than conventional consolidation/maintenance chemotherapy in all patients: final results of the International ALL Trial (MRC UKALL XII/ECOG E2993). Blood 2008;111:1827–33.^26^

**Figure 7:**
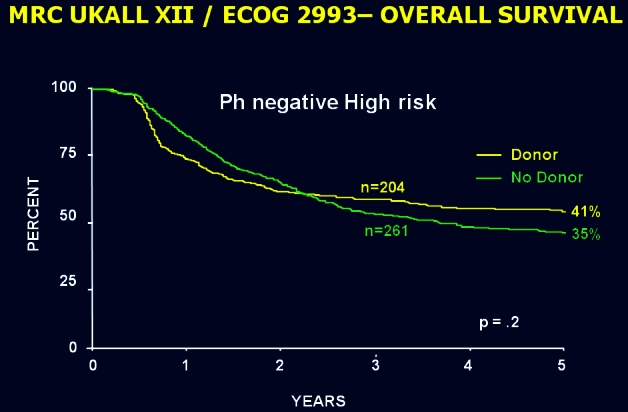
**Overall survival from diagnosis for donor versus no-donor for Ph-negative patients. Estimation of the effect of sibling donor transplant versus chemotherapy in high-risk patients.** Published with permission from Goldstone AH, et al. In adults with standard-risk acute lymphoblastic leukemia, the greatest benefit is achieved from a matched sibling allogeneic transplantation in first complete remission, and an autologous transplantation is less effective than conventional consolidation/maintenance chemotherapy in all patients: final results of the International ALL Trial (MRC UKALL XII/ECOG E2993). Blood 2008;111:1827–33.^26^

**Figure 8:**
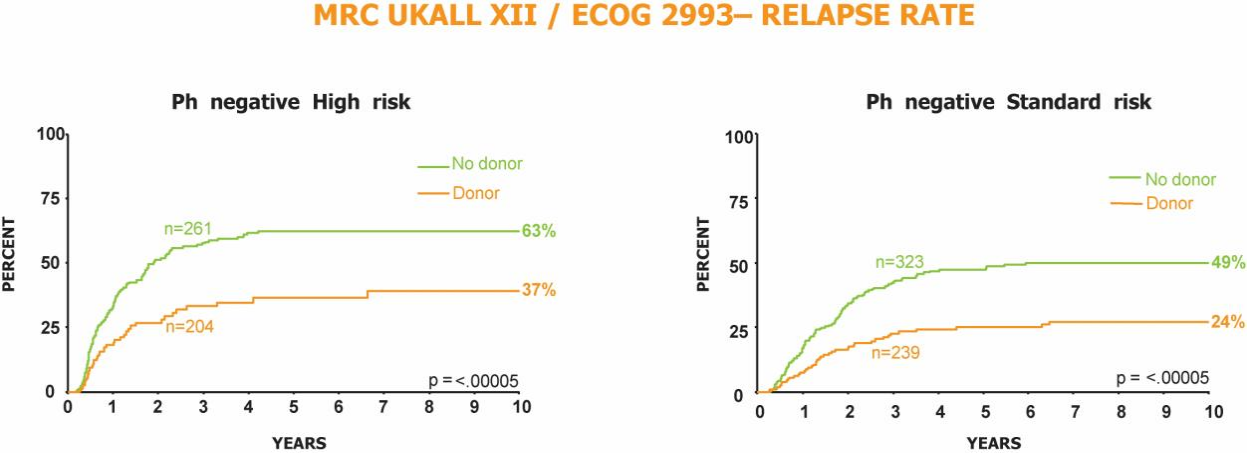
**Relapse rate for both high- and standard-risk patients is very significantly reduced among patients with a donor, the majority of whom underwent an allogeneic transplant.** Published with permission from Goldstone AH, et al. In adults with standard-risk acute lymphoblastic leukemia, the greatest benefit is achieved from a matched sibling allogeneic transplantation in first complete remission, and an autologous transplantation is less effective than conventional consolidation/maintenance chemotherapy in all patients: final results of the International ALL Trial (MRC UKALL XII/ECOG E2993). Blood 2008;111:1827–33.^26^

In contrast, the results of the large international ALL study surprised the international community by demonstrating, first, that standard-risk patients had a better outcome if offered an allogeneic transplant from a matched sibling in first complete remission (Figure 6) and, second, that high-risk patients, mostly those over the age of 35, had an unexpectedly high non-relapsed mortality that abrogated the superior benefit of allogeneic transplantation in this group (Figure 7). Prior to the results of this study, there had been a common perception that the well-known graft-versus-leukemia effect had only a minimal, if any, role in ALL. This study established, quite unequivocally, the very potent graft-versus-leukemia effect in ALL as demonstrated both in standard- and high-risk patients (Figure 8).

**Figure 9:**
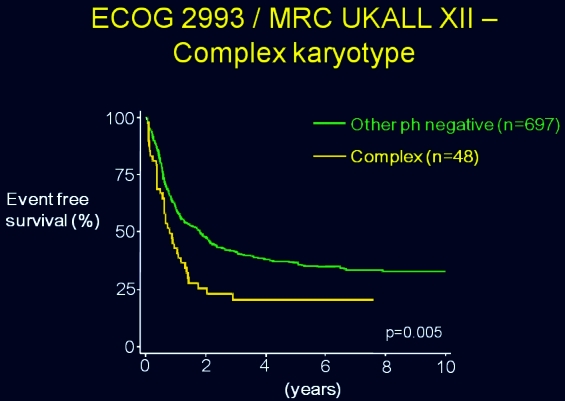
**Overall survival by cytogenetics: complex karyotype compared with all Philadelphia-chromosome-negative patients.** Published with permission from Moorman AV, et al. Karyotype is an independent prognostic factor in adult acute lymphoblastic leukemia (ALL): analysis of cytogenetic data from patients treated on the Medical Research Council (MRC) UKALLXII/Eastern Cooperative Oncology Group (ECOG) 2993 trial. Blood 2007;109:3189–97.^10^

Prior to 2005, there was little definitive information about cytogenetics in ALL. Although this had been accepted as being prognostically critical in AML, there was a paucity of information in ALL mostly due to the small number of patients in the studies. What had been mostly known was that the Philadelphia chromosome conferred a poor prognosis, but little else was confirmed. A complex karyotype in ALL was intuitively thought to portend a poor prognosis, as had been established in AML, but there had been no data to confirm this. This large international ALL study established, for the first time, the poor prognosis of patients with a complex karyotype (Figure 9),^10^ when compared with all other Philadelphia-chromosome-negative patients. *This transatlantic partnership confirmed the need and feasibility of large studies and emphasized the importance of collaboration among groups in uncommon disorders*.

## Bone Marrow Transplantation (BMT)

### Graft-versus-Host Disease

A careful examination of the literature in BMT is used to emphasize the need for care in assessing implications of newly published data.

Graft-versus-host disease (GvHD) had been the “scourge” of BMT, with mortality rates approaching 30%–40%, depending on typed donor and disease. It was known that GvHD is primarily initiated by donor T-cells, and thus, in the 1980s, investigators considered whether T-cell depletion could prevent or ameliorate GvHD. It was clear in the early 1980s that, despite technologies that were in place for successful T-cell depletion, the procedure itself carried formidable problems, mostly those of graft failure.^11^ It appeared that T-cells in the donor marrow were critical to maintain sustained engraftment, thus dampening the enthusiasm for this manipulation. In 1987, the first report of successful GvHD prevention, without graft failure, in human leukocyte antigen (HLA)-identical allogeneic bone marrow transplants was published using marrow that was depleted of T-cells by monoclonal antibodies and complement.^12^ In the same year, multiple results of successful T-cell depletion resulted in a short-lived euphoria when the problem of GvHD was thought to be “history.” The ink had virtually not dried on these papers when the excitement was dampened by reports in 1988 which pointed out an increased risk of relapse associated with T-cell depletion.^13^ In the subsequent year or two, multiple reports confirmed the early relapse post-allogeneic transplantation when T-cell depletion had been used.

**Figure 10:**
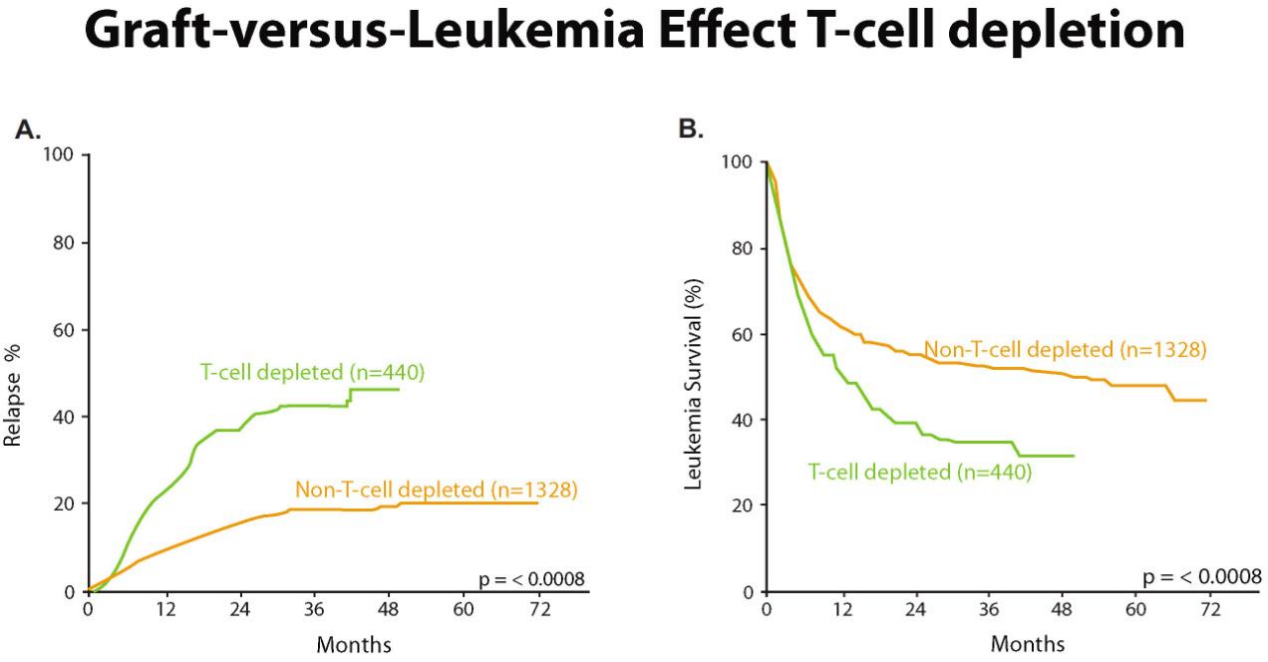
**(A) Probability of relapse after non-T-cell-depleted and T-cell-depleted HLA-identical sibling transplants for early intermediate leukemia. (B) Probability of leukemia-free survival (LFS) after non-T-cell-depleted and T-cell-depleted HLA-identical sibling transplants for early intermediate leukemia.** Published with permission from Marmont AM, et al. T-cell depletion of HLA-identical transplants in leukemia. Blood 1991;78:2120–30.^14^

A seminal experiment carried out in 1991 by Marmont in Italy^14^ demonstrated the markedly increased relapse among 440 T-cell-depleted patients compared with 1,328 non-T-cell-depleted patients with a parallel benefit in overall survival (Figure 10).

**Figure 11:**
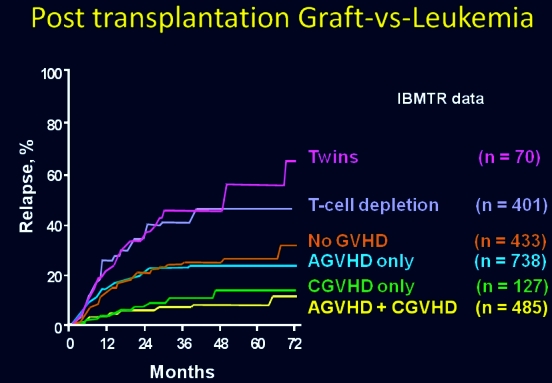
**Probability of relapse after bone marrow transplantation for early leukemia according to type of graft and development of GVHD.** Published with permission from Horowitz MM, et al. Graft-versus-leukemia reactions after bone marrow transplantation. Blood 1990;75(3):555–62.

The importance of the graft-versus-leukemia effect in humans has now been firmly established and was confirmed across a wide range of diseases in a classic paper summarizing data from the International Bone Marrow Transplant Registry (Figure 11). This retrospective registry study confirmed, in very large numbers, the increased relapse rate among syngeneic twins or patients undergoing T-cell depletions, compared with those experiencing acute or chronic GvHD, or both.^15^

### Timing of Bone Marrow Transplantation in Leukemia

**Figure 12:**
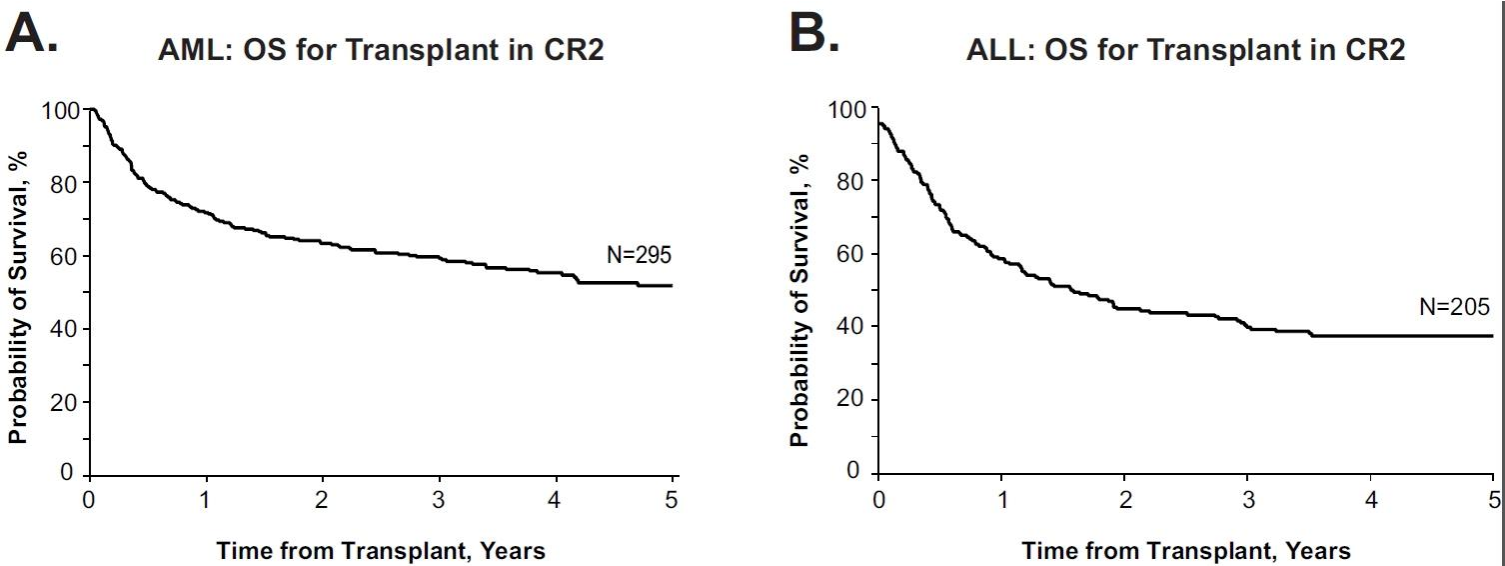
**Acute leukemia overall survival following second remission transplant.** Data from the Center for International Blood and Marrow Transplant Research (CIBMTR). Probability of survival after allogeneic hematopoietic stem cell transplantation with myeloablative conditioning for AML (A) or ALL (B) in CR2 in adults 18–50 years of age in the US, 2005–2007. Published with permission from Forman SJ and Rowe JM. The myth of the second remission of acute leukemia in the adult. Blood 2012 Dec 14. [Epub ahead of print].^18^

Allogeneic transplantation in first remission, in general, is recommended as the standard approach for patients at high risk for relapse with conventional therapy. Without doubt, allogeneic transplantation provides the most efficacious anti-leukemic therapy due to the potent graft-versus-leukemia (GVL) effect, and data have confirmed that allogeneic transplantation confers the lowest relapse rate for every subtype of AML. The high transplant-related morbidity and mortality is the only reason for not offering this to every patient with ALL or AML. In essence, this is a delicate balance between efficacy and toxicity.^16^ One of the most important issues relates to the timing of transplant. The foremost question among practitioners and patients is, given the high procedural mortality, should such a procedure be preferably reserved for patients in second complete remission or at relapse? Such considerations are bolstered by data demonstrating reasonable survival if transplant is performed in second remission (Figure 12). Given the high non-relapse mortality in allogeneic transplantation, such transplantation may sway patients away from transplant in first remission. While there is no doubt that allogeneic transplantation can be performed successfully in second complete remission, such reports are highly selective and confined to a small group of patients who have survived the relapse, achieved a second complete remission and were fit enough to undergo a trasnsplant, and for whom a donor was available. This represents a small minority of patients. If one considers the overall survival for all relapsed patients, this is no more than about 10%.^17^,^18^ Thus, presenting the optimistic data of second complete remission (CR2) to patients at diagnosis is thoroughly misleading and clearly needs to be avoided.

## INTENTION-TO-TREAT ANALYSES

Phase III studies, representing prospective randomized trials, are the gold standard, especially when analyzed by intention to treat. However, it is crucial to understand the limitations of such analyses. For example, phase III studies of transplantation usually underestimate the toxicity of the procedure because the donor arm is diluted by the number of patients who do not receive the transplant. They may also underestimate or overestimate efficacy depending on whether transplant is better than the comparator group. Furthermore, intention-to-treat analyses from diagnosis do not provide information for individual patients, as specified time points.

**Figure 13:**
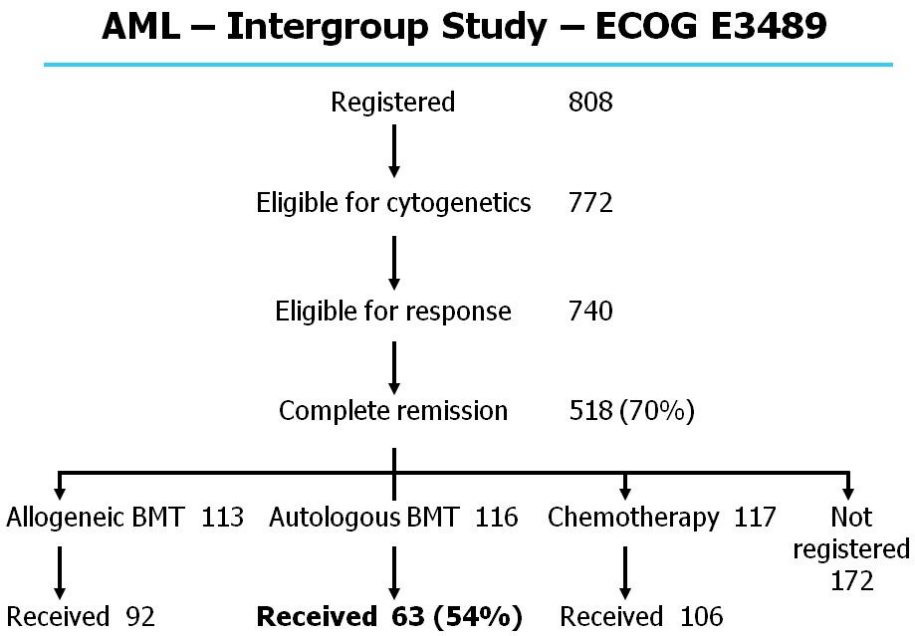
**US intergroup study prospectively evaluating various post-remission modalities.** It should be noted that among 116 patients eligible and actually randomized to an autologous transplant only 63 patients (54%) underwent this procedure. Data from Cassileth PA, NEJM.^27^

Importantly, a generic issue of transplant studies relates to the large number of patients who do not undergo the assigned or randomized procedure. Any intention-to-treat analysis can only be reliably assessed if patients actually receive the treatment specified in their assignment or randomization. This is notoriously so in autologous transplantation where as many as 50% of randomized patients do not receive their assigned randomized therapy (Figure 13). Although outcome curves are routinely published for such studies, based on intention-to-treat analyses, the true meaning is entirely unknown. Such data need to be interpreted with a great deal of circumspection.

## CONCLUSION

It is clear that when comparing studies differences in patient population, study conditions, study eligibility, and subtle differences in the conduct of a study all go towards emphasizing the lack of direct comparability across studies. It is crucial to be particularly careful in interpreting small studies and to be aware of early communication of data.

Lastly, even in well-conducted studies, it is vital to understand very carefully what large studies tell us and what they do not. The limitations of intention-to-treat analyses must be understood when considering published data. While good phase II data provide the backbone for further investigations, adequately sized, prospective phase III studies, conducted by a collaborative group of investigators, are the only way to move forward with definitive information.

## Figures and Tables

**Table 1:** **Phase II data—diffuse large cell lymphoma.** In the early 1980s, multiple new “improved” regimens.

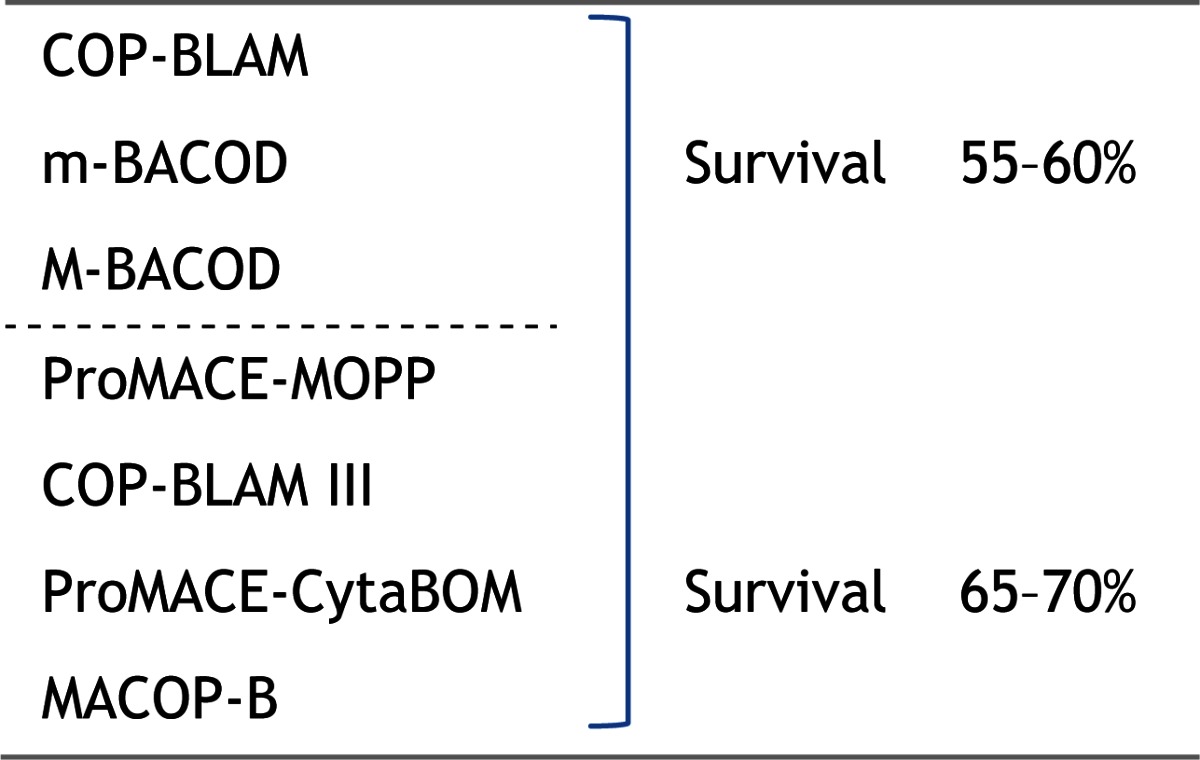

Many considered CHOP to be “unethical.”

**COP-BLAM**, cyclophosphamide, vincristine, prednisone, bleomycin, doxorubicin, and procarbazine; **m-BACOD**, bleomycin, doxorubicin, cyclophosphamide, vincristine, dexamethasone, methotrexate, and leucovorin; **M-BACOD**, methotrexate (high-dose) (with citrovorum factor rescue), bleomycin, doxorubicin, cyclophosphamide, vincristine and dexamethasone; **ProMACE–MOPP**, prednisone, methotrexate, doxorubicin cyclophosphamide, etoposide, mechlorethamine, vincristine, and procarbazine; **COP-BLAM III**, cyclophosphamide, infusional vincristine, prednisone, infusional bleomycin, doxorubicin, and procarbazine; **ProMACE-CytaBOM**, cyclophosphamide, doxorubicin, cytarabine, bleomycin, vincristine, methotrexate, and prednisone; **MACOP-B**, methotrexate, doxorubicin, cyclophosphamide, vincristine, prednisone, and bleomycin.

**Table 2:** **AML—induction therapy—3 days of anthracycline and 7 days of cytarabine (“3+7”).**

**Complete Remission (CR) Rate**	
SWOG	55–60%
ECOG	65–70%
CALGB	70–75%
MRC	75–85%

**CALGB**, the Cancer and Leukemia Group B; **ECOG**, Eastern Cooperative Oncology Group; **MRC**, Medical Research Council; SWOG, Southwest Oncology Group.

**Table 3:** **Varying criteria for definition of response in AML.**

**Group**	**Criteria**
SWOG	Central review at diagnosis **AND at CR**
ECOG	Central review at diagnosis **AND final CBC at ECOG-certified laboratory**
CALGB	Central review at diagnosis
MRC	Investigator report only

**CALGB**, **ECOG**, **MRC**, and **SWOG** as defined in Table 2; **CBC**, complete blood count; **CR**, complete remission.

**Table 4:** **Controlled trials of growth factors after induction therapy in AML.**

**Study**	***n***	**Reduction in Days to ANC 1000/μL**	**Documented Reduced Morbidity**

GM-CSF (sargramostim)			
Büchner, 1991	86	6–9	+
Rowe, 1995	117	6	+
**GM-CSF (molgrastim)**			
**Stone, 1995**	**379**	**2**	
Zittoun, 1996	53	-	
Lowenberg, 1997	316	5	
Witz, 1998	209	6	
Löfgren, 2004	110	8	
G-CSF (lenograstim)			
Dombert, 1995	173	6	+
Link, 1996	187	6	+
Goldstone, 2001	803	5	
Amadori, 2005	722	5	+
G-CSF (filgrastim)			
Ohno, 1990	67	12	+
Ohno, 1994	58	6	
Heil, 1997	521	5	+
Godwin, 1998	234	3–4	+
Usuki, 2002	270	6	+
Lehrnbecher, 2007	317	5	
Estey, 1994	197	13	

Published with permission from Rowe JM and Avivi I.^19^

**ANC**, absolute neutrophil count; **G-CSF**, granulocyte colony-stimulating factor; **GM-CSF**, granulocyte-macrophage colony-stimulating factor.

## Abbreviations:

ALL: acute lymphoblastic leukemia;
AML: acute myeloid leukemia;
BMT: bone marrow transplantation;
CALGB: the Cancer and Leukemia Group B;
CHOP: cyclophosphamide, doxorubicin, vincristine, and prednisone;
CIBMTR: Center for International Blood and Marrow Transplant Research;
COP-BLAM III: cyclophosphamide, infusional vincristine, prednisone, infusional bleomycin, doxorubicin, and procarbazine;
COP-BLAM: cyclophosphamide, vincristine, prednisone, bleomycin, doxorubicin, and procarbazine;
CR2: second complete remission;
ECOG: Eastern Cooperative Oncology Group;
G-CSF: granulocyte colony-stimulating factor;
GM-CSF: granulocyte-macrophage colony-stimulating factor;
GvHD: graft-versus-host disease;
GVL: graft-versus-leukemia;
HLA: human leukocyte antigen;
LFS: leukemia-free survival;
MACOP-B: methotrexate, doxorubicin, cyclophosphamide, vincristine, prednisone, and bleomycin;
M-BACOD: methotrexate (high-dose) (with citrovorum factor rescue), bleomycin, doxorubicin, cyclophosphamide, vincristine, and dexamethasone;
m-BACOD: bleomycin, doxorubicin, cyclophosphamide, vincristine, dexamethasone, methotrexate, and leucovorin;
MRC: Medical Research Council;
ProMACE-MOPP: prednisone, methotrexate, doxorubicin cyclophosphamide, etoposide, mechlorethamine, vincristine, and procarbazine;
ProMACE-CytaBOM: cyclophosphamide, doxorubicin, cytarabine, bleomycin, vincristine, methotrexate, and prednisone;
SWOG: Southwest Oncology Group.

## References

[1] McKelvey EM, Gottlieb JA, Wilson HE (1976). Hydroxyldaunomycin (Adriamycin) combination chemotherapy in malignant lymphoma. Cancer.

[2] Norton L, Simon R (1977). Tumor size, sensitivity to therapy, and design of treatment schedules. Cancer Treat Rep.

[3] Goldie JH, Coldman AJ, Gudauskas GA (1982). Rationale for the use of alternating non-cross-resistant chemotherapy. Cancer Treat Rep.

[4] Coleman M (1985). Chemotherapy for large-cell lymphoma: optimism and caution. Ann Intern Med.

[5] Fisher RI, Gaynor ER, Dahlberg S (1993). Comparison of a standard regimen (CHOP) with three intensive chemotherapy regimens for advanced non-Hodgkin's lymphoma. N Engl J Med.

[6] Coiffier B, Lepage E, Briere J (2002). CHOP chemotherapy plus rituximab compared with CHOP alone in elderly patients with diffuse large-B-cell lymphoma. N Engl J Med.

[7] Rowe JM (2012). The impact of mutational profiling on AML prognosis. Best Pract Res Clin Haematol.

[8] Stone RM, Berg DT, George SL (1995). Granulocyte-macrophage colony-stimulating factor after initial chemotherapy for elderly patients with primary acute myelogenous leukemia. Cancer and Leukemia Group B. N Engl J Med.

[9] Thomas X, Boiron JM, Huguet F (2004). Outcome of treatment in adults with acute lymphoblastic leukemia: analysis of the LALA-94 trial. J Clin Oncol.

[10] Moorman AV, Harrison CJ, Buck GA (2007). Karyotype is an independent prognostic factor in adult acute lymphoblastic leukemia (ALL): analysis of cytogenetic data from patients treated on the Medical Research Council (MRC) UKALLXII/Eastern Cooperative Oncology Group (ECOG) 2993 trial. Blood.

[11] Martin PJ, Hansen JA, Buckner CD (1985). Effects of in vitro depletion of T cells in HLA-identical allogeneic marrow grafts. Blood.

[12] Herve P, Cahn JY, Flesch M (1987). Successful graft-versus-host disease prevention without graft failure in 32 HLA-identical allogeneic bone marrow transplantations with marrow depleted of T cells by monoclonal antibodies and complement. Blood.

[13] Goldman JM, Gale RP, Horowitz MM (1988). Bone marrow transplantation for chronic myelogenous leukemia in chronic phase. Increased risk for relapse associated with T-cell depletion. Ann Intern Med.

[14] Marmont AM, Horowitz MM, Gale RP (1991). T-cell depletion of HLA-identical transplants in leukemia. Blood.

[15] Horowitz MM, Gale RP, Sondel PM (1990). Graft-versus-leukemia reactions after bone marrow transplantation. Blood.

[16] Cornelissen JJ, Gratwohl A, Schlenk RF (2012). The European LeukemiaNet AML Working Party consensus statement on allogeneic HSCT for patients with AML in remission: an integrated-risk adapted approach. Nat Rev Clin Oncol.

[17] Fielding AK, Richards SM, Chopra R (2007). Outcome of 609 adults after relapse of acute lymphoblastic leukemia (ALL); an MRC UKALL12/ECOG 2993 study. Blood.

[18] Forman SJ, Rowe JM (2012). The myth of the second remission of acute leukemia in the adult. Blood.

[19] Rowe JM, Avivi I, Hoffman R, Furie B, McGlave P (2009). Clinical use of Hematopoietic Growth Factors. Hematology: Basic Principles and Practice.

[20] Philip T, Guglielmi C, Hagenbeek A (1995). Autologous bone marrow transplantation as compared with salvage chemotherapy in relapses of chemotherapy-sensitive non-Hodgkin's lymphoma. N Engl J Med.

[21] Moore JO, George SL, Dodge RK (2005). Sequential multiagent chemotherapy is not superior to high-dose cytarabine alone as postremission intensification therapy for acute myeloid leukemia in adults under 60 years of age: Cancer and Leukemia Group B Study 9222. Blood.

[22] Fernandez HF, Sun Z, Yao X (2009). Anthracycline dose intensification in acute myeloid leukemia. N Engl J Med.

[23] Buchner T, Berdel WE, Haferlach C (2009). Age-related risk profile and chemotherapy dose response in acute myeloid leukemia: a study by the German Acute Myeloid Leukemia Cooperative Group. J Clin Oncol.

[24] Petersdorf S, Kopecky K, Stuart RK (2009). Preliminary results of Southwest Oncology Group Study S0106: an international intergroup phase 3 randomized trial comparing the addition of gemtuzumab ozogamicin to standard induction therapy versus standard induction therapy followed by a second randomization to post-consolidation gemtuzumab ozogamicin versus no additional therapy for previously untreated acute myeloid leukemia. Blood.

[25] Burnett AK, Hills RK, Milligan D (2011). Identification of patients with acute myeloblastic leukemia who benefit from the addition of gemtuzumab ozogamicin: results of the MRC AML15 trial. J Clin Oncol.

[26] Goldstone AH, Richards SM, Lazarus HM (2008). In adults with standard-risk acute lymphoblastic leukemia, the greatest benefit is achieved from a matched sibling allogeneic transplantation in first complete remission, and an autologous transplantation is less effective than conventional consolidation/maintenance chemotherapy in all patients: final results of the International ALL Trial (MRC UKALL XII/ECOG E2993). Blood.

[27] Cassileth PA, Harrington DP, Appelbaum FR (1998). Chemotherapy compared with autologous or allogeneic bone marrow transplantation in the management of acute myeloid leukemia in first remission. N Engl J Med.

